# Longitudinal associations between body mass index, physical activity, and healthy dietary behaviors in adults: A parallel latent growth curve modeling approach

**DOI:** 10.1371/journal.pone.0173986

**Published:** 2017-03-15

**Authors:** Youngdeok Kim, Jung-Min Lee, Jungyoon Kim, Emily Dhurandhar, Ghada Soliman, Nizar K. Wehbi, James Canedy

**Affiliations:** 1 Department of Kinesiology and Sport Management, Texas Tech University, Lubbock, Texas, United States of America; 2 School of Health, Physical Education and Recreation, University of Nebraska Omaha, Omaha, Nebraska, United States of America; 3 College of Public Health, University of Nebraska Medical Center, Omaha, Nebraska, United States of America; 4 Department of Environmental, Occupational and Geospatial Health Sciences, City University of New York, New York, New York, United States of America; 5 Nebraska Medicine, Omaha, Nebraska, United States of America; Universita degli Studi Magna Graecia di Catanzaro Scuola di Medicina e Chirurgia, ITALY

## Abstract

**Background:**

Physical activity (PA) and healthy dietary behaviors (HDB) are two well-documented lifestyle factors influencing body mass index (BMI). This study examined 7-year longitudinal associations between changes in PA, HDB, and BMI among adults using a parallel latent growth curve modeling (LGCM).

**Methods:**

We used prospective cohort data collected by a private company (SimplyWell LLC, Omaha, NE, USA) implementing a workplace health screening program. Data from a total of 2,579 adults who provided valid BMI, PA, and HDB information for at least 5 out of 7 follow-up years from the time they entered the program were analyzed. PA and HDB were subjectively measured during an annual online health survey. Height and weight measured during an annual onsite health screening were used to calculate BMI (kg·m^2^). The parallel LGCMs stratified by gender and baseline weight status (normal: BMI<25, overweight BMI 25–29.9, and obese: BMI>30) were fitted to examine the longitudinal associations of changes in PA and HDB with change in BMI over years.

**Results:**

On average, BMI gradually increased over years, at rates ranging from 0.06 to 0.20 kg·m^2^·year, with larger increases observed among those of normal baseline weight status across genders. The increases in PA and HDB were independently associated with a smaller increase in BMI for obese males (b = -1.70 and -1.98, respectively), and overweight females (b = -1.85 and -2.46, respectively) and obese females (b = -2.78 and -3.08, respectively). However, no significant associations of baseline PA and HDB with changes in BMI were observed.

**Conclusions:**

Our study suggests that gradual increases in PA and HDB are independently associated with smaller increases in BMI in overweight and obese adults, but not in normal weight individuals. Further study is warranted to address factors that check increases in BMI in normal weight adults.

## Introduction

Obesity is a growing public health concern that affects nearly one-third of the US adult population [1,2] A large body of literature demonstrates the negative impact of obesity on the increased risk of adverse health outcomes such as Type II Diabetes, cardiovascular diseases, and all-causes of mortality among adults [3,4]. The prevalence of obesity has significantly increased during the last several decades [1,5] and is projected to continue increasing with 65 million more adults with obesity in the US in 2030 than in 2010 [6]. Furthermore, medical costs attributed to the treatment of obesity-related morbidity are projected to rise to more than $22–66 billion per year by 2030 [6], which collectively increases the societal and public health burden imposed by obesity.

Obesity is a preventable chronic disease that is strongly associated with lifestyle behaviors contributing to energy balance [7]. Physical activity (PA) and diet are the modifiable lifestyle behaviors that play significant roles in achieving and maintaining energy balance for both treatment and prevention of obesity [7,8]. For instance, healthy dietary behaviors (HDB), such as consuming whole grain products, low-fat foods, and plant proteins, helps to avoid excessive energy intake [9,10], whereas increases in PA increase energy expenditure [11]. A large body of literature exists demonstrating effective lifestyle intervention strategies for the treatment of obesity [8,12]. However, relatively little attention has been given to exploring preventive roles of PA and HDB in reducing obesity risk, in part due to the lack of longitudinal observations [13,14], upon which to base the development of proactive prevention strategies. Furthermore, attempts thus far to examine longitudinal associations of PA and HDB with respect to the risk of obesity have often analyzed the two factors separately or do not concurrently adjust for the potential time-varying confounding effects of the respective behaviors [14–16]. In addition, although PA is the main predictor of successful weight loss maintenance [17,18], the preventive effect of PA on long-term weight gain has sometimes been inconsistent [16,19], leading to confusion and controversy over the role of PA in reducing the risk of obesity [20].

Given the naturally occurring weight-gain with age in the adult population [21], the societal burden for the treatment of obesity [8], and the difficulty of achieving and maintaining weight loss [22], a more proactive approach may be to understand the preventive roles of PA and HDB on obesity risk based on longitudinal observations. Thus, this study examined the 7-year longitudinal associations of PA and HDB with body mass index (BMI), one of the most widely used indicators of obesity, in a large sample of adults. We employed a rigorous analytical method, parallel latent growth curve modeling (LGCM), to estimate 1) average changes in PA, HDB, and BMI; and 2) the extent to which changes in PA and HDB explain the change in BMI over a 7-year period, while controlling for time-varying confounding effects of the respective behaviors.

## Methods

### Survey data and study sample

Data for this study came from a private company (SimplyWell LLC, Omaha, NE) that offers personalized healthcare management services to employees at more than 100 organizations across various industries such as agriculture, information technology, marketing/business general, health care, and factory labor in Nebraska, USA. The services include both onsite and online educational/counseling programs promoting healthy lifestyle behaviors based on results from annual health screenings, which include both onsite clinical examinations and laboratory tests, and online health surveys. A detailed description of the services can be found at https://secure.simplywell.com/view/public/index.xhtml. All data were de-identified before submission to the investigators, and Institutional Review Board approval (IRB# 209-14-NH—University of Nebraska Medical Center) was waived for our study’s non-invasive, secondary data analysis.

A total of 22,885 employees participated in the annual health screening from 2004 through 2013. From this group, we selected cohorts who had initially enrolled during 2004 through 2007 (n = 17,315; average follow-up years of 3.27 (SD = 2.62)). Cohort members were further excluded if they: 1) were pregnant or diagnosed with severe chronic diseases (e.g., cancers) or disorders that limit daily activities (n = 182); 2) failed to participate in the annual health screenings more than 4 times during the 7 consecutive years after entering (n = 14,536); or 3) did not provide valid responses to the demographic characteristics at entry (n = 18). Thus, the final analytic sample consisted of 2,579 adults (1,072 male) with an average age of 42.72 (SD = 10.08) and provided valid data for 7 consecutive years. The baseline characteristics of the final sample were on average, 1 year older, more likely to be Caucasian, and less likely to smoke, or be obese, and had higher levels of income, education, PA, and HDB compared to those who were excluded (*P*’s < .05).

### Assessments of physical activity and healthy dietary behaviors

As part of the screening program, the participants were asked to complete the annual online health risk questionnaire during the enrollment years. PA and HDB were measured using three self-reported, Likert-type questions asking about 1) aerobic exercise; 2) general PA status; and 3) strength exercise, and 1) fat intake; 2) breads and grains consumption; and 3) protein intake, respectively. The specific questions and response categories of each question can be found in Table A in S1 File.

A principal component analysis was performed for each set of PA and HDB questions across 7 consecutive years, in order to calculate the single dimension, principal component scores (PCS) representing the extent to which participants were physically active and had HDB, respectively. The first principal component was retained based on the eigenvalue >1 criterion and scree-plot examination. The total variance (%) of PCSs explained by the set of PA and HDB questions ranged from 70.90% to 72.13% for PA, and from 54.63% to 57.61% for HDB, respectively (Table B in S1 File). Thus final outcome variables of PA and HDB across each year were the PCS_(PA)_ and PCS_(HDB)_, standardized to a mean of zero and a standard deviation of one. Higher PCSs indicated a greater tendency to be physically active and to have better HDB, respectively.

### Assessments of body mass index and other variables

During the annual onsite health screenings, trained staff collected standardized measurements of each participant’s height (cm) and weight (kg). BMI (kg·m^2^) was calculated as weight divided by the square of height across each measurement year. Individual BMI at entry year was used to categorize the participants into normal (<25 kg/m^2^), overweight (25–29.9 kg·m^2^), and obese (≥30 kg·m^2^) status at the baseline.

Baseline demographic characteristics were also obtained via an online health survey at entry year. They included age (years), race/ethnicity (Non-Hispanic white, Others), education levels (≤high school, some college, college graduate, graduate degree), family income (<$40k, $40–59.9k, $60k-$79.9k, ≥$80k), smoking (yes, no), and alcohol consumption (average number of alcoholic beverage drinks per day in the past two weeks).

### Statistical analyses

All data analyses were stratified by gender (male and female) and baseline weight status (normal, overweight, and obese) to obtain gender- and weight status-specific parameter estimates. Descriptive statistics for baseline characteristics of the study sample were calculated. One-way analysis of variance (ANOVA) with Tukey’s pairwise comparisons was used to examine the mean difference in continuous variables by weight status. For nominal (race/ethnicity and smoking) and ordinal (education levels and family income) categorical variables, the *x*^2^ test of independence and Mantel-Haenszel *x*^*2*^ test of linear association were used, respectively, to examine the associations of each categorical variable with weight status.

**Fig 1:**
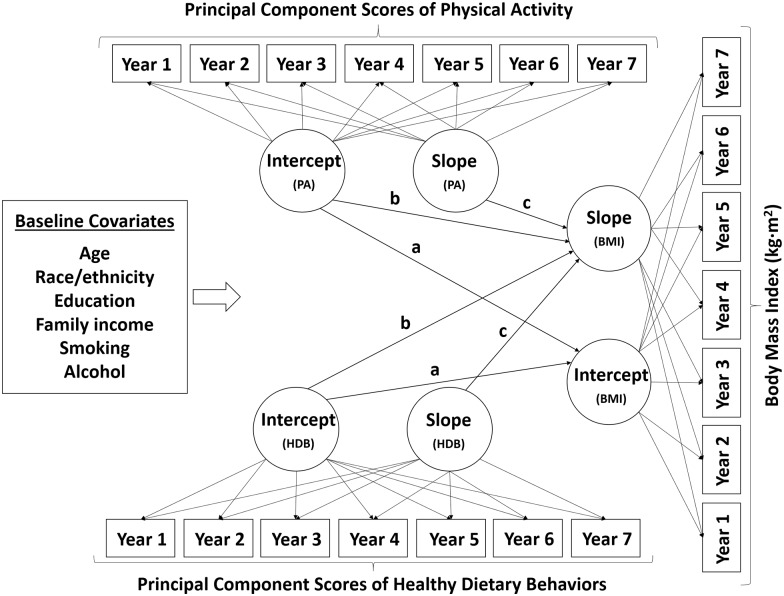
A schematic diagram depicting the parallel latent growth curve model controlling for baseline covariates. ‘a’ denotes the paths examining the cross-sectional associations of baseline PA and HDB with baseline BMI (Intercept_(PA)_ → Intercept_(BMI)_; Intercept_(HDB)_ → Intercept_(BMI)_). ‘b’ denotes the paths examining the prospective associations of baseline PA and HDB with change in BMI (Intercept_(PA)_ → Slope_(BMI)_; Intercept_(HDB)_ → Slope_(BMI)_). ‘c’ denotes the parallel associations of changes in PA and HDB with change in BMI (Slope_(PA)_ → Slope_(BMI)_; Slope_(HDB)_ → Slope_(BMI)_).

Latent growth curve modeling (LGCM) was employed to examine the longitudinal trajectories of PA, HDB, and BMI over the 7-year period. LGCM is a multivariate statistical method within the framework of structural equation modeling that allows for modeling of repeated measures data to estimate inter-individual heterogeneity in intra-individual growth patterns over time [23]. The simplest unconditional LGCM for an outcome *y*_*ti*_ of individual *i* at time *t* is presented as: *y*_*ti*_ = *λ*_*0t*_ *η*_*0i*_ + *λ*_*1t*_ *η*_*1*i_ + *ε*_*ti*_, where *η*_*0i*_ = *ν*_*0*_ + *ζ*_*0i*_ and *η*_*1i*_ = *ν*_*1*_ + *ζ*_*1i*_. The outcome (*y*_*ti*_) is predicted by two latent growth factors, *η*_*0i*_ (i.e., intercept) and *η*_*1*i_ (i.e., slope) with the expected means of *ν*_*0*_ and *ν*_*1*_ and associated residuals of *ζ*_*0i*_ and *ζ*_*1i*_, respectively, and a time-specific residual *ε*_*ti*_. For this study, we established a set of conditional LGCMs for each outcome variable (PA, HDB, and BMI) in that the latent growth factors are predicted by a set of baseline covariates *x*_*M*_: *η*_*0i*_ = *ν*_*0*_ + *γ*_*01*_ *x*_*1i*_ + *γ*_*02*_ *x*_*2i*_ +… + *γ*_*0M*_ *x*_*Mi*_ + *ζ*_*0i*_ and *η*_*1i*_ = *ν*_*1*_ + *γ*_*11*_ *x*_*1i*_ + *γ*_*12*_ *x*_*2i*_ +… + *γ*_*1M*_ *x*_*Mi*_ + *ζ*_*1i*_, including age, race, education, family income, smoking status, and alcohol consumption. The factor loadings, *λ*_*0t*_ and *λ*_*1t*_, on the respective latent factors were constrained to [1, 1, 1, 1, 1, 1, 1] and [0, 1, 2, 3, 4, 5, 6], respectively, to estimate latent growth parameters representing the baseline level of outcome variable (i.e., intercept) and the rate of annual change in outcome variable (i.e., slope), respectively. We compared the model-data-fits between the LGCM models with and without a latent quadratic growth factor to test the non-linearity of growth rates of outcome variables across gender and weight status. Overall, the LGCM with the latent quadratic growth factor generally showed better model-data-fits when comparing to the linear LGCM; however, the latent quadratic growth factor was significant only for the BMI growth model among normal weight female and the HDB growth model among overweight male. Further the model-data-fits of linear LGCM were acceptable for all outcome variables across gender and weight status, and thus, we retained the linear LGCM as a global measurement model for the sake of simplicity and comparability. Finally, the parallel LGCM was established predicting latent growth factors of BMI using the latent growth factors of PA and HDB while controlling for baseline covariates as well as the time-varying effects of the respective behaviors (Fig 1). Specific parameters tested in the model included 1) cross-sectional associations between BMI, PA, and HDB at baseline (Intercept_(PA)_ → Intercept_(BMI)_; and Intercept_(HDB)_ → Intercept_(BMI)_); 2) prospective associations of baseline PA and HDB with the change in BMI (Intercept_(PA)_ → Slope_(BMI)_; and Intercept_(HDB)_ → Slope_(BMI)_); and 3) parallel associations of changes in PA and HDB with change in BMI over 7-year period (Slope_(PA)_ → Slope_(BMI)_; and Slope_(HDB)_ → Slope_(BMI)_). The general interpretations of latent growth parameters (i.e., intercept and slope) for LGCM and specific parameter estimates (cross-sectional, prospective, and parallel associations) from the parallel LGCM can be found in Table B in S1 File.

The model-data-fit of each LGCM was evaluated based on a comparative fit index (CFI), Tucker-Lewis index (TLI), and root mean square error of approximation (RMSEA). The model was considered to fit the data adequately if CFI ≥.90, TLI ≥.90, and RMSEA ≤.08 [24]. A full information maximum likelihood estimation was used for all LGCM analyses to account for missing data, assuming missing at random, using the Mplus v7.2 (Muthén & Muthén, LA, CA).

## Results

Descriptive statistics for baseline characteristics of the study sample by gender and weight status are presented in Table 1. The majority of male participants were overweight (49.4%) or obese (29.8%) and a large percentage of female participants (44.0%) were normal weight at the time of entry into the program. In general, the participants were characterized by a large percentage of non-Hispanic white (87.5% - 93.8%), and relatively high education and family income levels (approximately more than 50% are college graduate with a family income ≥ $60k). The levels of self-reported PA and HDB represented by PCS were significantly different by weight status, in that, on average, the obese group had lowest levels of PA and HDB for both genders.

**Table 1:** Baseline characteristics of study sample by genders and weight status ^a^.

	Male (N = 1,072)				Female (N = 1,507)			
	Weight status			P-value ^b^	Weight status			P-value ^b^
	Normal	Overweight	Obese		Normal	Overweight	Obese	
	(n = 223, 20.8%)	(n = 530, 49.4%)	(n = 319, 29.8%)		(n = 663, 44.0%)	(n = 405, 26.9%)	(n = 439, 29.1%)	
Age, years	41.2 (9.8)	44.5 (9.7)	44.0 (9.0)	< .001 ^m^^,^^n^	40.1 (10.6)	43.1 (10.3)	43.9 (9.6)	< .001 ^m^^,^^n^
Race/ethnicity				0.052				< .001
Non-Hispanic White	188 (90.1%)	450 (84.9%)	288 (90.3%)		622 (93.8%)	360 (88.9%)	384 (87.5%)	
Others	35 (9.9%)	80 (15.1%)	31 (9.72%)		41 (6.2%)	45 (11.1%)	55 (12.5%)	
Education				< .001				< .001
≤ High school	39 (17.5%)	98 (18.5%)	65 (20.4%)		70 (10.6%)	43 (10.6%)	70 (16.0%)	
Some college	29 (13.0%)	119 (22.5%)	82 (25.7%)		123 (18.6%)	112 (27.7%)	142 (32.4%)	
College graduate	104 (46.6%)	242 (45.7%)	142 (44.5%)		375 (56.6%)	189 (46.7%)	184 (41.9%)	
Graduate degree	51 (22.9%)	71 (13.4%)	30 (9.4%)		95 (14.3%)	61 (15.1%)	43 (9.8%)	
Family income				0.003				< .001
< $40k	44 (19.7%)	78 (14.7%)	37 (11.6%)		122 (18.4%)	73 (18.0%)	117 (26.7%)	
$40k-< $60k	41 (18.4%)	87 (16.4%)	49 (15.4%)		148 (22.3%)	82 (20.3%)	124 (28.3%)	
$60k-< $80k	44 (19.7%)	129 (24.3%)	70 (21.9%)		116 (17.5%)	94 (23.2%)	88 (20.1%)	
≥ $80k	94 (42.2%)	236 (44.5%)	163 (51.1%)		277 (41.8%)	156 (38.5%)	110 (25.1%)	
Smoke				0.716				0.162
Yes	31 (13.9%)	63 (11.9%)	42 (13.2%)		61 (9.2%)	41 (10.1%)	56 (12.8%)	
No	192 (86.1%)	467 (88.1%)	277 (86.8%)		602 (90.8%)	364 (89.9%)	383 (87.2%)	
Alcohol ^c^	3.0 (3.2)	3.4 (3.7)	2.6 (3.1)	0.004 ^n^	2.1 (2.4)	2.1 (2.8)	1.2 (2.0)	< .001 ^m^
Physical activity								
Aerobic exercise ^d^	2.3 (2.0)	2.1 (2.0)	1.5 (1.8)	< .001 ^m^	2.6 (2.0)	2.4 (2.0)	1.7 (1.8)	< .001 ^m^
General PA status ^e^	3.6 (1.3)	3.5 (1.3)	3.1 (1.3)	< .001 ^m^	3.8 (1.3)	3.5 (1.4)	2.8 (1.3)	< .001 ^l^^,^^m^^,^^n^
Strength exercise ^f^	2.1 (1.3)	2.0 (1.2)	1.6 (1.0)	< .001 ^m^	2.1 (1.2)	1.9 (1.2)	1.5 (1.0)	< .001 ^l^^,^^m^^,^^n^
PCS_(PA)_ ^g^	0.13 (1.0)	0.01 (1.0)	-0.34 (0.9)	< .001 ^m^	0.25 (1.0)	0.07 (1.0)	-0.40 (0.9)	< .001 ^l^^,^^m^^,^^n^
Healthy dietary behaviors								
Fat intake ^h^	3.1 (0.9)	3.0 (0.8)	2.8 (0.9)	< .001 ^m^	3.5 (0.7)	3.4 (0.8)	3.0 (0.8)	< .001 ^l^^,^^n^
Bread and grains ^i^	3.0 (1.0)	2.9 (1.0)	2.9 (1.0)	0.562	3.1 (1.0)	3.1 (0.1)	2.8 (1.0)	< .001 ^l^^,^^n^
Protein intake ^j^	2.2 (0.8)	2.1 (0.8)	2.0 (0.8)	0.006 ^l^^,^^m^	2.3 (0.9)	2.2 (0.8)	2.0 (0.8)	< .001 ^l^^,^^n^
PCS_(HDB)_ ^k^	-0.05 (1.0)	-0.19 (1.0)	-0.37 (1.0)	< .001 ^n^	0.20 (0.9)	0.08 (0.9)	-0.28 (1.0)	< .001 ^l^^,^^n^

PA, physical activity; PCS, principle component scores; HDB, healthy dietary behaviors.

^a^ mean (SD) for continuous variables and n (%) for categorical variables.

^b^ *P* value was obtained from one-way analysis of variance with Tukey pairwise comparisons for continuous variables, *x*^2^ test of independence for nominal categorical variables, and Mantel-Haenszel *x*^*2*^ test of linear association for ordinal categorical variables (education and family income), respectively.

^c^ average number of alcoholic beverage drinks per day in the past two weeks.

^d^ “how many days per week do you engage in aerobic exercise of at least 20 to 30 minute duration (e.g., fitness walking, cycling, jogging, swimming, aerobic dance, active sport)?” with 8-point Likert type of scale.

^e^ “mark the response that best describes your current activity level” with 6-point Likert type of scale.

^f^ “how many times per week do you do strength building exercises such as sit-ups, pushups, or use weight training equipment?” with 4-point Likert type of scale.

^g^ 1^st^ principal component score with a mean of zero and a standard deviation of one, explained by PA items among total sample.

^h^ 5-point Likert type of scale ranged from “nearly always eat high fat foods” to “eat only low fat foods”.

^i^ 5-point Likert type of scale ranged from “nearly always eat refined grain products” to “eat only whole grain products”.

^j^ 5-point Likert type of scale ranged from “nearly always eat animal proteins” to “eat only vegetable proteins”.

^k^ 1^st^ principal component score with a mean of zero and a standard deviation of one, explained by HDB items among total sample.

^l^ signifcantly different between normal and overweight in Tukey’s pairwise comparisons.

^m^ signifcantly different between normal and obese in Tukey’s pairwise comparisons.

^n^ signifcantly different between overweight and obese in Tukey’s pairwise comparisons.

Table 2 presents the model-data fit indices for the gender-and weight status-specific LGCMs established for each outcome variable (BMI, PA, HDB) as well as for the parallel LGCM. In general, the longitudinal changes in BMI, PA, and HDB over the 7-year period after adjusting for baseline covariates were well identified from the respective LGCM across gender and weight status with acceptable model-data fits (RMSEA (≤.08), CFI (≥.90), and TLI (≥.90)).

**Table 2:** The model-data fit indices for the latent growth curve models by gender and weight status.

	Male				Female			
	*x*^2^(*df*)	RMSEA (90% CI)	CFI	TLI	*x*^2^(*df*)	RMSEA (90% CI)	CFI	TLI
LGCM ^a^								
Outcome: BMI								
Normal	138.4 (73) ^c^	0.063 (0.047, 0.079)	0.94	0.92	181.0 (73) ^c^	0.047 (0.039, 0.056)	0.98	0.97
Overweight	178.7 (73) ^c^	0.052 (0.043, 0.062)	0.95	0.94	252.9 (73) ^c^	0.078 (0.068, 0.089)	0.89	0.87
Obese	174.4 (73) ^c^	0.066 (0.053, 0.079)	0.96	0.95	121.7 (73) ^c^	0.039 (0.026, 0.051)	0.98	0.98
Outcome: PCS_(PA)_								
Normal	85.0 (73)	0.027 (0.000, 0.049)	0.99	0.99	133.7 (73) ^c^	0.035 (0.026, 0.045)	0.98	0.97
Overweight	85.7 (73)	0.018 (0.000, 0.032)	0.99	0.99	99.4 (73) ^d^	0.030 (0.012, 0.044)	0.98	0.98
Obese	103.5 (73) ^d^	0.036 (0.018, 0.051)	0.96	0.96	102.8 (73) ^d^	0.030 (0.015, 0.044)	0.97	0.97
Outcome: PCS_(HDB)_								
Normal	115.3 (73) ^c^	0.051 (0.032, 0.068)	0.95	0.94	104.9 (73) ^c^	0.026 (0.013, 0.036)	0.99	0.98
Overweight	92.7 (73)	0.023 (0.000, 0.035)	0.99	0.99	67.5 (73)	0.000 (0.000, 0.024)	1.00	1.00
Obese	79.1 (73)	0.016 (0.000, 0.037)	0.99	0.99	110.1 (73) ^c^	0.034 (0.020, 0.047)	0.97	0.97
Parallel LGCM ^b^								
Normal	574.9 (362) ^c^	0.051 (0.043, 0.059)	0.93	0.92	804.7 (362) ^c^	0.043 (0.039, 0.047)	0.96	0.95
Overweight	645.0 (362) ^c^	0.038 (0.034, 0.043)	0.95	0.95	817.2 (362) ^c^	0.056 (0.051, 0.061)	0.90	0.87
Obese	719.4 (362) ^c^	0.056 (0.050, 0.062)	0.92	0.91	874.4 (362) ^c^	0.057 (0.052, 0.062)	0.92	0.90

BMI, body mass index (kg/m2); CFI, comparative fit index; CI, confidence interval; *df*, degree of freedom; HDB, healthy dietary behaviors; LGCM, latent growth curve model; PA, physical activity; RMSEA, root mean square error of approximation; TLI, Tucker-Lewis index.

^a^ gender- and weight status-specific LGCM for each outcome variable after controlling for baseline covariates including age, race, education, family income, smoking status, and alcohol consumption.

^b^ final model integrating three LGCMs for each outcome variable while controlling for baseline covariates including age, race, education, family income, smoking status, and alcohol consumption.

^c^ *P* < 0.01.

^d^ *P* < 0.05.

Latent growth parameters estimated from each LGCM (Table 3) demonstrated significant increases in BMI per year (Slope_(BMI)_) for both genders. On average, annual increases in BMI for males and females were 0.18 kg·m^2^·year and 0.20 kg·m^2^·year for normal, 0.11 kg·m^2^·year and 0.17 kg·m^2^·year for overweight, and 0.06 kg·m^2^ and 0.07 kg·m^2^ for obese weight status groups, respectively. There was significant covariance between latent growth parameters for normal (Cov_(BMI)_ = 0.09 and 0.14 for male and female) and overweight (Cov_(BMI)_ = 0.14 and 0.20 for male and female) groups, indicating a positive linear relationship between baseline BMI and annual changes in BMI in these groups. There were significant annual increases in the levels of PA in overweight and obese males (Slope_(PA)_ = 0.02 and 0.03, respectively) and HDB in obese males (Slope_(HDB)_ = 0.03), whereas no significant changes in PA and HDB were observed in females across weight status groups over the years. The covariance between latent growth parameters of PA (Cov_(PA)_) and HDB (Cov_(HDB)_) were negatively estimated for all groups, indicating negative linear relationships of baseline levels of PA and HDB with annual changes in PA and HDB, respectively.

**Table 3:** Estimated mean and 95% confidence intervals of latent growth parameters ^a^.

	Weight status					
	Normal		Overweight		Obese	
	Estimate	95% CI	Estimate	95% CI	Estimate	95% CI
Male						
Outcome: BMI (kg·m^2^)						
Intercept_(BMI)_	23.46 ^c^	23.24, 23.68	27.49 ^c^	27.37, 27.61	33.32 ^c^	32.95, 33.69
Slope_(BMI)_	0.18 ^c^	0.14, 0.22	0.11 ^c^	0.07, 0.15	0.06 ^d^	0.02, 0.10
Cov_(BMI)_ ^b^	0.09 ^c^	0.03, 0.15	0.14 ^c^	0.10, 0.18	0.15	-0.01, 0.31
Outcome: PCS_(PA)_						
Intercept_(PA)_	0.16 ^f^	0.04, 0.28	0.01	-0.07, 0.09	-0.31 ^c^	-0.41, -0.21
Slope_(PA)_	-0.01	-0.03, 0.01	0.02 ^c^	0.00, 0.04	0.03 ^c^	0.01, 0.05
Cov_(PA)_ ^b^	-0.03 ^c^	-0.05, -0.01	-0.03 ^c^	-0.05, -0.01	-0.04 ^c^	-0.06, -0.02
Outcome: PCS_(HDB)_						
Intercept_(HDB)_	-0.06	-0.18, 0.06	-0.14 ^c^	-0.22, -0.06	-0.34 ^c^	-0.44, -0.24
Slope_(HDB)_	-0.01	-0.03, 0.01	0.01	-0.01, 0.03	0.03 ^c^	0.01, 0.05
Cov_(HDB)_ ^b^	-0.01	-0.03, 0.01	-0.01	-0.03, 0.01	-0.02	-0.04, 0.00
Female						
Outcome: BMI						
Intercept_(BMI)_	22.31 ^c^	22.17, 22.45	27.33 ^c^	27.17, 27.49	35.73 ^c^	35.26, 36.2
Slope_(BMI)_	0.20 ^c^	0.18, 0.22	0.17 ^c^	0.13, 0.21	0.07 ^d^	-0.01, 0.15
Cov_(BMI)_ ^b^	0.14 ^c^	0.08, 0.20	0.20 ^c^	0.12, 0.28	-0.04	-0.37, 0.29
Outcome: PCS_(PA)_						
Intercept_(PA)_	0.28 ^c^	0.20, 0.36	0.06	-0.04, 0.16	-0.36 ^c^	-0.44, -0.28
Slope_(PA)_	-0.002	-0.02, 0.02	0.002	-0.02, 0.02	0.004	-0.02, 0.02
Cov_(PA)_ ^b^	-0.03 ^c^	-0.05, -0.01	-0.04 ^c^	-0.06, -0.02	-0.03 ^c^	-0.05, -0.01
Outcome: PCS_(HDB)_						
Intercept_(HDB)_	0.23 ^c^	0.17, 0.29	0.12 ^c^	0.04, 0.20	-0.23 ^c^	-0.31, -0.15
Slope_(HDB)_	0.001	-0.02, 0.02	-0.01	-0.03, 0.01	0.01	-0.01, 0.03
Cov_(HDB)_ ^b^	-0.01	-0.03, 0.01	-0.03 ^c^	-0.05, -0.01	-0.02 ^d^	-0.04, 0.00

BMI, body mass index (kg/m2); CI, confidence interval; Cov, covariance; PA, physical activity; PCS: principal component scores; HDB, healthy dietary behaviors.

^a^ all parameters were estimated from the gender- and BMI level-specific LGCMs after adjusting baseline covariates including age, race, education, family income, smoking status, and alcohol consumption. Interpretations of parameter estimates can be found in Table C in S1 File.

^b^ covariance between intercept and slope

^c^ *P*< 0.01

^d^ *P*< 0.05

Table 4 presents the results of the parallel LGCM. After controlling for baseline covariates, significantly negative cross-sectional associations of PA with BMI at baseline (Intercept_(PA)_ → Intercept_(BMI)_) were observed in obese males (b = -1.51) and overweight and obese females (b = -0.37 and -1.83, respectively). Meanwhile, the baseline level of HDB was not significantly associated with BMI at baseline (Intercept_(HDB)_ → Intercept_(BMI)_) across gender or weight status. With respect to the prospective associations of baseline levels of PA and HDB with the change in BMI over the 7-year period, we found no significant associations across gender or weight status, with the exception of overweight males in that significantly positive association was observed between baseline level of HDB and average change in BMI (Intercept_(HDB)_ → Slope_(BMI)_; b = 0.06).

**Table 4:** Parameter estimates from the parallel latent growth curve model ^a^.

	Weight status					
	Normal		Overweight		Obese	
	Estimate (b) ^b^	95% CI	Estimate (b) ^b^	95% CI	Estimate (b) ^b^	95% CI
Male						
Cross-sectional association ^c^						
Intercept_(PA)_ → Intercept_(BMI)_	-0.08	-0.39, 0.23	-0.08	-0.29, 0.12	-1.51 ^f^	-2.23, -0.79
Intercept_(HDB)_ → Intercept_(BMI)_	-0.17	-0.49, 0.16	0.08	-0.14, 0.29	0.23	-0.43, 0.88
Prospective association ^d^						
Intercept_(PA)_ → Slope_(BMI)_	-0.02	-0.07, 0.04	-0.03	-0.08, 0.02	0.05	-0.05, 0.14
Intercept_(HDB)_ → Slope_(BMI)_	0.04	-0.02, 0.10	0.06 ^g^	0.00, 0.11	-0.07	-0.15, 0.02
Parallel association ^e^						
Slope_(PA)_ → Slope_(BMI)_	-0.50	-1.09, 0.10	-0.45	-1.08, 0.18	-1.70 ^f^	-2.66, -0.73
Slope_(HDB)_ → Slope_(BMI)_	-0.49	-1.41, 0.44	-0.84 ^g^	-1.69, 0.01	-1.98 ^g^	-3.77, -0.18
Female						
Cross-sectional association ^c^						
Intercept_(PA)_ → Intercept_(BMI)_	-0.03	-0.26, 0.19	-0.37 ^f^	-0.61, -0.14	-1.83 ^f^	-2.71, -0.95
Intercept_(HDB)_ → Intercept_(BMI)_	-0.22	-0.48, 0.04	-0.25	-0.52, 0.02	-0.58	-1.38, 0.22
Prospective association ^d^						
Intercept_(PA)_ → Slope_(BMI)_	-0.01	-0.05, 0.04	-0.04	-0.11, 0.03	0.05	-0.08, 0.18
Intercept_(HDB)_ → Slope_(BMI)_	-0.04	-0.09, 0.02	0.04	-0.04, 0.13	0.06	-0.06, 0.18
Parallel association ^e^						
Slope_(PA)_ → Slope_(BMI)_	-0.75 ^f^	-1.20, -0.31	-1.85 ^f^	-2.75, -0.94	-2.78 ^f^	-4.09, -1.46
Slope_(HDB)_ → Slope_(BMI)_	-0.52	-1.23, 0.20	-2.46 ^f^	-3.57, -1.36	-3.08 ^f^	-4.77, -1.38

BMI, body mass index (kg/m2); CI, confidence interval; PA, physical activity; HDB, healthy dietary behaviors.

^a^ the model was adjusted for baseline covariates including age, race, education, family income, smoking status, and alcohol consumption. Interpretations of parameter estimates can be found in Table D in S1 File.

^b^ the estimates are unstandardized regression coefficients (b)

^c^ the estimates can be interpreted as changes in baseline BMI (kg·m^2^·year) by one unit changes in baseline PCS_(PA)_ or PCS_(HDB)_

^d^ the estimates can be interpreted as changes in the growth rate of BMI (kg·m^2^·year) by one unit changes in baseline PCS_(PA)_ or PCS_(HDB)_

^e^ the estimates can be interpreted as changes in the growth rate of BMI (kg·m^2^·year) by one unit changes in growth rates of PCS_(PA)_ or PCS_(HDB)_

^f^ *P* < 0.01

^g^ *P* < 0.05

Finally, the significant parallel associations of changes in PA and HDB with change in BMI were observed in obese males (b = -1.70 for Slope_(PA)_ → Slope_(BMI)_; and b = -1.98 for Slope_(HDB)_ → Slope_(BMI)_), and in overweight females (b = -1.85 for Slope_(PA)_ → Slope_(BMI)_; and b = -2.46 for Slope_(HDB)_ → Slope_(BMI)_) and obese females (b = -2.78 for Slope_(PA)_ → Slope_(BMI)_; and b = -3.08 for Slope_(HDB)_ → Slope_(BMI)_). For overweight males, only the change in HDB was significantly associated with change in BMI (b = -0.84), while the change in PA was the only factor associated with change in BMI for females of normal weight (b = -0.75).

## Discussion

Obesity is a global epidemic that is largely attributed to energy imbalance. PA and HDB are the two most influential lifestyle behaviors that help maintain energy balance; however, the evidence regarding the preventive effects of those behaviors on reducing the risk of obesity is equivocal [16,19]. As part of our continuing efforts to improve understanding of the roles of PA and HDB in overcoming the obesity epidemic, we took an advantage of a large 7-year prospective cohort to examine the longitudinal associations of PA and HDB with BMI in adults.

The findings of our current study highlight parallel associations of changes in PA and HDB with the change in BMI over time, but the significance of the observed associations varies by gender and baseline weight status. The increase in PA was independently associated with smaller increases in BMI for obese males; while for females, the associations were consistently observed regardless of baseline weight status, with greater effects observed as baseline BMI increases. Meanwhile, the change in HDB was shown to be a significant predictor of change in BMI for both overweight and obese participants regardless of gender, but not for those with normal baseline weight status.

The finding that associations of PA and HDB with BMI gain over time were more pronounced in those with a higher baseline weight status is an intriguing finding from our study. These behaviors were not associated with prevention of BMI gain in normal weight individuals, with the exception of increases in PA preventing BMI gain in normal weight females. In fact, our findings call into question the idea that the development of overweight and obesity in normal weight individuals can be prevented by improved HDB in either males or females, and increased PA for males, especially considering that initially normal weight males experienced the most weight gain. Our findings do, however, underscore the importance of these behaviors as particularly helpful for lessening the burden of obesity for those already struggling with obesity and overweight. They moreover highlight the need for further research on effective strategies to prevent weight gain in normal weight individuals, and affirm the idea that many factors beyond PA and HDB contribute to energy balance and may play a role in weight gain [25].

Our findings are in part consistent with previous studies that showed favorable associations of PA and HDB in preventing long-term weight gain particularly in overweight and obese adults. For example, a 15-year follow-up study [26] examined the longitudinal associations between walking patterns and weight change in 4,995 adults, and demonstrated that an increase in walking over years was significantly associated with less weight gain particularly in women with heavier baseline weight (≥75^th^ percentile), whereas no statistically significant association was observed for males with lighter baseline weight (<25^th^ percentile). Another study examined the 9-year change in BMI in relation to changes in food patterns among 33,840 Swedish women and showed greatest effects of increasing healthy eating patterns on reducing BMI over years in obese women when compared to normal and overweight women [27].

As mentioned earlier, however, there have been equivocal reports on the longitudinal associations of PA and HDB with BMI or weight gain, with some studies showing little or no associations, and other studies reporting the associations but in different groups. For instance, the findings from a recent study [28] involving 1,231 Australian adults demonstrated no relation between subjectively measured diet quality and 15-year changes in any anthropometric measures including waist circumference and BMI in females after controlling for energy misreporting in addition to other covariates such as baseline age, occupation, PA, smoking, alcohol, etc., whereas lower increases in BMI and waist circumference were associated with a high-quality diet in males who were in compliance with the Australian dietary guidelines. Another longitudinal study of 2,436 Danish adults [29] reported no persistent relationship between changes in food intake patterns and BMI over up to 10 years of observation. Specifically, the study demonstrated that factor scores representing different food intake patterns from 26-item food frequency questionnaire were not prospectively nor longitudinally associated with changes in BMI over 5- and 11-year follow-ups. On the other hand, Lee et al [14], who examined the prospective associations between PA and long-term weight gains in 34,079 healthy US women, observed an inverse dose-response relation between PA and weight gain over 3-year periods for women with normal baseline weight status, whereas no relation was found for overweight and obese women.

Although direct comparisons across studies are complicated due to methodological variations, one factor that may explain apparent discrepancies between our findings and those of others could be related to differences in analytical methodology used to quantify the longitudinal associations among variables [30]. A majority of studies described above used the levels of PA or HDB at either baseline or a specific time point to predict prospective changes in anthropometric measures or weight status (i.e., obesity) in later years. Thus, their implications may not relate to the longitudinal associations, but rather to prospective associations predicting future events based on prior levels of exposure to given variable. The analytical approach used in our study, parallel process LGCM, overcomes such limitations and concurrently estimated three types of associations (cross-sectional, prospective, and parallel) while controlling for the confounding effects of repeated measures of respective variables. We found no significant prospective associations of baseline PA and HDB with the change in BMI across gender and baseline weight status; whereas the increases in PA and HDB were significantly associated with the change in BMI over years. These findings are generally aligned with the results of the systematic review conducted by Fogelholm and Kukkonen-Harjula [19] who reported that studies which examined the change in PA as an exposure variable demonstrated more consistent findings with greater increase in PA being associated with less weight gain over years, whereas the findings from studies using the baseline PA in predicting long-term weight change were controversial. Alternatively, discrepancies between our findings and the findings of other studies that find no relationship between PA and HDB with changes in BMI or weight may also be due to our sample. Our sample was from a company implementing a worksite wellness program, thus, we are unable to control for any intervention effects in this sample that may explain some of the associations we observed. Nonetheless, our findings do provide some insight as to the impact of HDB and PA in this setting.

Longitudinal changes in PA and HDB in our analytic sample varied by gender and baseline weight status (Table 3). Particularly, overweight and obese males reported increased levels of PA and HDB (only obese males) over years; whereas no significant changes were observed for the remaining groups. These findings may imply that levels of PA and HDB at baseline are, on average, constantly maintained over years, which could suggest that higher PA and HDB at baseline may be continued over years. However, the results of covariance analysis between latent growth parameters (intercept vs. slope) consistently demonstrated negative associations, indicating that the greater levels of PA and HDB at baseline are associated with smaller increases or larger decreases in PA and HDB over years. On the other hand, our analytic sample showed a gradual increase in BMI over time, ranging from 0.06 to 0.20 kg·m^2^·year, with greater increases observed among those with normal baseline weight status across genders. These increases are similar to those observed in previous studies supporting natural weight gain with age in adults [16,27,31]; however, our analysis also found positive covariance between latent growth parameters in the BMI growth model, particularly for both normal- and overweight groups at baseline, indicating that a higher BMI at baseline accelerates the increase in BMI over times in those groups. Although the annual increase in BMI was seemingly small, even a small amount of increase in BMI may cause a number of long-term adverse health consequences [32,33]. Since the subsequent changes in BMI were inversely associated with changes in PA and HDB, but not with baseline levels of PA and HDB, future intervention strategies to prevent long-term weight gain may consider focusing on promoting sustainable PA and HDB over longer periods.

The unique strengths of this study include its findings generated from a rigorous analytical method using longitudinal data with a relatively large sample size. Particularly, unlike previous studies that calculated changes in outcome variables from two time points (i.e., changes between baseline and end of follow-up period), the LGCM established in our study modeled repeated observations of outcome variables across 7 years which allowed us to fully account for within-person variability when estimating longitudinal changes in each outcome variable. Furthermore, as described above, the parallel LGCM integrated three LGCMs for each outcome variable and produced unbiased estimates of the cross-sectional, prospective, and parallel associations among variables.

The present study nonetheless has several limitations that should be considered when interpreting the results. First, the present findings were based on data from the employees who were participating in a workplace wellness program, which offers online educational/counseling services along with annual health screenings. Therefore our findings might be influenced by intervention effects, particularly for the estimated annual changes in outcome variables in Table 3, and the estimates of prospective associations in Table 4. However, the parallel associations of changes in PA and HDB with changes in BMI over years estimated from the parallel LGCM are not influenced by intervention effects, as they estimate the extent to which the increase or decrease in PA or HDB is associated with an increase or decrease in BMI over years, regardless of whether the estimated changes in PA, HDB, and BMI were induced by any intervention effects or not. Second, the majority of our sample were non-Hispanic whites with relatively high socio-economic status. Considering that race/ethnicity and socio-economic status are the two of most frequently cited contextual factors influencing health behaviors [8], our results may not be valid for generalization to other population segments of different race/ethnicity and socio-economic status. Third, although our analyses are based on LGCM adjusted for baseline BMI in estimating annual changes in BMI, they may reflect effects of regression to the mean on BMI outcomes due to the subgroup analyses by baseline weight status. Finally, the psychometric properties of PA and HDB questionnaires used in the annual health risk survey portion of the workplace wellness program have not been systematically examined. Although they were constructed by a team of doctoral-level experts at SimplyWell LLC (Omaha, NE, USA) and reliability of measures in current data are provided in Table B in S1 File, there is no of empirical evidence regarding the questionnaire’s convergent and/or criterion validity or their sensitivity to measuring changes in PA and HDB over periods of time. In addition, the outcome variables of PA and HDB were the PCA scores calculated from three questions representing different aspects of PA and HDB, respectively (aerobic exercise, general PA, and strength exercise for PA; and fat intake, breads and grains consumption, protein intake for HDB), which are not sufficient to capture all aspects of PA and HDB influencing energy balance. Furthermore, PCA scores only provide information about the relative levels of PA and HDB of individual given sample data, and thus, our interpretations of PCA scores were limited as to the extent to which the person was physically active or had HDB relative to sample mean. Collectively, although subjectively measured PA and HDB are preferred in large observational studies due to their administrative advantages, future study is encouraged to use objective measures of PA and HDB in order to obtain more accurate estimates of energy expenditure and intake. Particularly, the use of objectively measured PA and HDB would facilitate absolute classification of the individual based upon current PA and diet recommendations to establish the long-term, dose-response effects on obesity risk.

## Conclusions

In summary, our data add to the body of literature examining the longitudinal associations of PA and HDB with BMI in adults. The findings of the current study demonstrate inverse, parallel associations of PA and HDB with BMI over time. The increases in PA and HBD were independently associated with smaller increases in BMI particularly for those who were overweight and obese, but not for those who were normal weight at baseline. However, there were no consistent cross-sectional nor prospective associations observed across genders and baseline weight status. Our findings imply that consistent improvements in PA and HDB may lessen the burden of obesity in those who are already overweight or obese. Future research should focus on alternative, more cause-specific prevention strategies, or more intensive changes in PA or HDB to determine optimal doses for prevention of overweight and obesity in normal weight individuals.

## Supporting information

S1 File Table A. Survey Questions for Physical Activity and Healthy Diet Behaviors. Table B. The Results of Principal Component Analysis for Physical Activity and Health Dietary Behaviors Questions. Table C. Interpretations of Parameter Estimates from the Latent Growth Curve Models. Table D. Interpretations of Parameter Estimates from the parallel Latent Growth Curve Model.
(DOCX)

## Abbreviations

BMI: Body mass index
CFI: Comparative fit index
CI: Confidence interval
HDB: Healthy dietary behaviors
LGCM: Latent growth curve model
PA: Physical activity
RMSEA: Root mean square error of approximation
TLI: Tucker-Lewis index
