# Supplementary material for: Longitudinal associations between body mass index, physical activity, and healthy dietary behaviors in adults: A parallel latent growth curve modeling approach
Source: PLoS One. 2017 Mar 15;12(3):e0173986. doi: 10.1371/journal.pone.0173986 (PMC5352028; doi:10.1371/journal.pone.0173986)
Supplement: S1 File — Table A. Survey Questions for Physical Activity and Healthy Diet Behaviors. Table B. The Results of Principal Component Analysis for Physical Activity and Health Dietary Behaviors Questions. Table C. Interpretations of Parameter Estimates from the Latent Growth Curve Models. Table D. Interpretations of Parameter Estimates from the parallel Latent Growth Curve Model. (DOCX) [file pone.0173986.s001.docx]

| Domains | Questions | Response categories |
| --- | --- | --- |
| **Physical activity** | | |
| Aerobic exercise | How many days per week do you engage in aerobic exercise of at least 20 to 30 minutes duration (e.g., fitness walking, cycling, jogging, swimming, aerobic dance, active sport)? | 0 (no exercise program) to 7 (seven days per week) |
| General physical activity status | Mark the response that best describes your current activity level | 0 - No exercise program; 1 - I have no regular exercise program, generally avoid walking or exertion when possible; 2 - I occasionally walk for pleasure or exercise sufficiently to cause heavy breathing or perspiration (sweat); 3 - I get regular exercise in work or recreation requiring modest physical activity such as golf, yard work, calisthenics, weight lifting, or table tennis, up to 1 hour per week; 4 - I get regular exercise in work or recreation requiring modest physical activity such as golf, yard work, calisthenics, weight lifting, or table tennis, more than 1 hour per week; 5 - I participate regularly in more active physical exercise such as brisk walking, jogging, swimming, cycling, rowing, active sports like tennis or handball |
| Strength exercise | How many times per week do you do strength building exercises such as sit-ups, pushups, or use weight training equipment? | 1 – None; 2 - Once a week; 3 - Twice a week; and 4 - Three plus time weekly |

Appendix Table A. Survey Questions for Physical Activity and Healthy Dietary Behaviors

Appendix Table A. Survey Questions for Physical Activity and Healthy Diet Behaviors (continue)

| Domains | Questions | Response categories |
| --- | --- | --- |
| **Healthy diet behaviors** | | |
| Fat intake | Indicate the kinds of foods you usually eat [**High fat examples**: hamburgers, hot dogs, bologna, steaks, sour cream, cheese, whole milk, eggs, butter, cake, pastry, ice cream, chocolate, fried foods and many fast foods; **Low fat examples**: lean meats, skinless poultry, fish, skim milk, low fat dairy products, fruit desserts, gelatin, pasta, legumes (peas and beans)] | 0 (nearly always eat high fat foods), 1 (eat mostly high fat foods), 2 (eat both about the same), 3 (eat mostly low fat foods), 4 (eat primarily low fat foods), and 5 (eat only low fat foods) |
| Bread and grains consumption | Indicate the kinds of protein foods you usually eat [**Refined grain examples**: white bread, rolls, regular pancakes and waffles, white rice, typical breakfast cereals, typical baked goods; **Whole grain examples**: whole grain breads, brown rice, oatmeal, whole grain or high fiber cereals] | 0 (nearly always eat refined grain products), 1 (eat mostly refined grain products), 2 (eat both about the same), 3 (eat mostly whole grain products), 4 (eat primarily whole grain products), and 5 (eat only whole grain products) |
| Protein intake | Indicate the kinds of protein foods you usually eat [**Animal sources**: meats, poultry, fish, cheese, eggs; **Vegetable sources**: legumes (peas, beans, lentils), tofu, soy meats, nut foods, veggie burger, vegetarian entrees] | 0 (nearly always eat animal proteins), 1 (eat mostly animal proteins), 2 (eat both about the same), 3 (eat mostly vegetable proteins), 4 (eat primarily vegetable proteins), and 5 (eat only vegetable proteins) |

Appendix Table B. The Results of Principal Component Analysis for Physical Activity and Healthy Dietary Behavior Questions

|  | Component loadings of 1^st^ principal component ^a^ | | | | | | |
| --- | --- | --- | --- | --- | --- | --- | --- |
|  | Year 1 | Year 2 | Year 3 | Year 4 | Year 5 | Year 6 | Year 7 |
| **Physical activity** |  |  |  |  |  |  |  |
| Q1 - Aerobic exercise | 0.88 | 0.88 | 0.88 | 0.88 | 0.89 | 0.88 | 0.88 |
| Q2 - General physical activity | 0.84 | 0.85 | 0.86 | 0.85 | 0.85 | 0.85 | 0.86 |
| Q3 - Strength exercise | 0.81 | 0.80 | 0.80 | 0.79 | 0.80 | 0.80 | 0.80 |
| Total variance explained (%) | 70.90 | 71.16 | 72.08 | 71.21 | 71.70 | 71.73 | 72.13 |
| Cronbach's α | 0.77 | 0.77 | 0.79 | 0.79 | 0.78 | 0.78 | 0.78 |
| Valid sample (n) | 2522 | 2025 | 2232 | 2261 | 2289 | 2223 | 1981 |
| **Healthy dietary behaviors** |  |  |  |  |  |  |  |
| Q1 - Fat intake | 0.80 | 0.80 | 0.79 | 0.78 | 0.79 | 0.80 | 0.80 |
| Q2 - Bread and grains consumption | 0.77 | 0.76 | 0.79 | 0.75 | 0.75 | 0.75 | 0.75 |
| Q3 - Protein intake | 0.69 | 0.67 | 0.70 | 0.69 | 0.67 | 0.68 | 0.70 |
| Total variance explained (%) | 56.92 | 55.40 | 57.61 | 55.07 | 54.63 | 55.49 | 56.19 |
| Cronbach's α | 0.62 | 0.60 | 0.63 | 0.61 | 0.60 | 0.60 | 0.60 |
| Valid sample (n) | 2544 | 2046 | 2264 | 2307 | 2320 | 2278 | 2013 |

^a^ 1^st^ principal component was retained based on the eigenvalues >1 criterion and scree plot examination

Appendix Table C. Interpretations of Parameter Estimates from the Latent Growth Curve Models

| Parameters | Interpretations |
| --- | --- |
| **Outcome: BMI** | BMI unit: kg/m^2^ |
| Intercept | Average BMI at baseline |
| Slope | Average annual change (positive or negative) in BMI |
| Cov_(BMI)_ | Association between intercept (average BMI at baseline) and slope (average annual change in BMI):  When the slope is positive:  1) Positive covariance – higher BMI at baseline and larger increases in BMI per year  2) Negative covariance – higher BMI at baseline and smaller increases in BMI per year  When the slope is negative:  1) Positive covariance – higher BMI at baseline and smaller decreases in BMI per year  2) Negative covariance – higher BMI at baseline and larger decreases in BMI per year |
| **Outcome: HDB** | HDB unit: Principal component scores (PCS) with a mean of 0 and standard deviation of 1 |
| Intercept | Average PCS_(HDB)_ at baseline |
| Slope | Average annual change (positive or negative) in PCS_(HDB)_ |
| Cov_(HDB)_ | Association between intercept (average PCS_(HDB)_ at baseline) and slope (average annual change in PCS_(HDB)_):  When the slope is positive:  1) Positive covariance – higher PCS_(HDB)_ at baseline and larger increases in PCS_(HDB)_ per year  2) Negative covariance – higher PCS_(HDB)_ at baseline and smaller increases in PCS_(HDB)_ per year  When the slope is negative:  1) Positive covariance – higher PCS_(HDB)_ at baseline and smaller decreases in PCS_(HDB)_ per year  2) Negative covariance – higher PCS_(HDB)_ at baseline and larger decreases in PCS_(HDB)_ per year |

Appendix Table C. Interpretations of Parameter Estimates from the Latent Growth Curve Models (continued)

| Parameters | Interpretations |
| --- | --- |
| **Outcome: PA** | PA unit: Principal component scores (PCS) with a mean of 0 and standard deviation of 1 |
| Intercept | Average PCS_(PA)_ at baseline |
| Slope | Average annual change (positive or negative) in PCS_(PA)_ |
| Cov_(PA)_ | Association between intercept (average PCS_(PA)_ at baseline) and slope (average annual change in PCS_(PA)_):  When the slope is positive:  1) Positive covariance – higher PCS_(PA)_ at baseline and larger increases in PCS_(PA)_ per year  2) Negative covariance – higher PCS_(PA)_ at baseline and smaller increases in PCS_(PA)_ per year  When the slope is negative:  1) Positive covariance – higher PCS_(PA)_ at baseline and smaller decreases in PCS_(PA)_ per year  2) Negative covariance – higher PCS_(PA)_ at baseline and larger decreases in PCS_(PA)_ per year |

Appendix Table D. Interpretations of Parameter Estimates from the Parallel Latent Growth Curve Model

| Parameter | Interpretations |
| --- | --- |
| **Cross-sectional associations** |  |
| Intercept_(PA)_ → Intercept_(BMI)_ | Association between PCS_(PA)_ and BMI (kg/m^2^) at baseline |
| Intercept_(HDB)_ → Intercept_(BMI)_ | Association between PCS_(HDB)_ and BMI (kg/m^2^) at baseline |
| **Prospective associations** |  |
| Intercept_(PA)_ → Slope_(BMI)_ | Association between PCS_(PA)_ at baseline and annual change in BMI (kg/m^2^):  When the Slope_(BMI)_ in Table 3 is positive:  1) Positive association - higher baseline PCS_(PA)_ and larger increases in BMI (kg/m^2^) per year  2) Negative association - higher baseline PCS_(PA)_ and smaller increases in BMI (kg/m^2^) per year  When the Slope_(BMI)_ in Table 3 is negative:  1) Positive association - higher baseline PCS_(PA)_ and smaller decreases in BMI (kg/m^2^) per year  2) Negative association - higher baseline PCS_(PA)_ and larger decreases in BMI (kg/m^2^) per year |
| Intercept_(HDB)_ → Slope_(BMI)_ | Association between PCS_(HDB)_ at baseline and annual change in BMI (kg/m^2^):  When the Slope_(BMI)_ in Table 3 is positive:  1) Positive association - higher baseline PCS_(HDB)_ and larger increases in BMI (kg/m^2^) per year  2) Negative association - higher baseline PCS_(HDB)_ and smaller increases in BMI (kg/m^2^) per year  When the Slope_(BMI)_ in Table 3 is negative:  1) Positive association - higher baseline PCS_(HDB)_ and smaller decreases in BMI (kg/m^2^) per year  2) Negative association - higher baseline PCS_(HDB)_ and larger decreases in BMI (kg/m^2^) per year |

Appendix Table D. Interpretations of Parameter Estimates from the Parallel Latent Growth Curve Model (continued)

| Parameter | Interpretations |
| --- | --- |
| **Parallel associations** |  |
| Slope_(PA)_ → Slope_(BMI)_ | Association between annual changes in PCS_(PA)_ and annual changes in BMI (kg/m^2^):  When the Slope_(BMI)_ in Table 3 is positive:  1) Positive association - higher Slope_(PA)_ and larger increases in BMI (kg/m^2^) per year  2) Negative association - higher Slope_(PA)_ and smaller increases in BMI (kg/m^2^) per year  When the Slope_(BMI)_ in Table 3 is negative:  1) Positive association - higher Slope_(PA)_ and smaller decreases in BMI (kg/m^2^) per year  2) Negative association - higher Slope_(PA)_ and larger decreases in BMI (kg/m^2^) per year |
| Slope_(HDB)_ → Slope_(BMI)_ | Association between annual changes in PCS_(HDB)_ and annual changes in BMI (kg/m^2^):  When the Slope_(BMI)_ in Table 3 is positive:  1) Positive association - higher Slope_(HDB)_ and larger increases in BMI (kg/m^2^) per year  2) Negative association - higher Slope_(HDB)_ and smaller increases in BMI (kg/m^2^) per year  When the Slope_(BMI)_ in Table 3 is negative:  1) Positive association - higher Slope_(HDB)_ and smaller decreases in BMI (kg/m^2^) per year  2) Negative association - higher Slope_(HDB)_ and larger decreases in BMI (kg/m^2^) per year |
